# Selection of host plants for production of *Clanis bilineata* (Lepidoptera: Sphingidae)

**DOI:** 10.1371/journal.pone.0303017

**Published:** 2024-06-24

**Authors:** Xiaofeng Li, Mingming Guo, Kebin Li, Song Li, Honglin Feng, Jiwei Fan

**Affiliations:** 1 Lianyungang Academy of Agricultural Sciences, Lianyungang, China; 2 State Key Laboratory for Biology of Plant Diseases and Insect Pests, Institute of Plant Protection, Chinese Academy of Agricultural Sciences, Beijing, China; 3 Yichang Agricultural Product Quality and Safety Supervision and Testing Station, Yichang, China; 4 Department of Entomology, Louisiana State University Agricultural Center, Baton Rouge, LA, United States of America; Government College University Faisalabad, PAKISTAN

## Abstract

*Clanis bilineata* Walker (Lepidoptera: Sphingidae), a burgeoning edible insect, is experiencing rising demand in China and other regions. Despite this interest, larval production is currently constrained by the limitations of artificial production technologies, particularly the selection of optimal host plants. This study rigorously evaluated the performance of *C*. *bilineatha* larvae on four main host plants: round-leaf soybean, pointed-leaf soybean, black locust, and kudzu. Preference tests demonstrated that the larvae were most attracted to black locust (34.76 ± 4.65%), with subsequent preferences for kudzu (25.00 ± 6.12%), round-leaf soybean (23.17 ± 2.79%), and pointed-leaf soybean (14.02 ± 4.74%). No significant preference differences were noted between round-leaf soybean and either black locust or kudzu. In feeding assays, the larvae exhibited a marked preference for round-leaf soybean (37.36 ± 0.81 g, total feeding amount for larvae), followed by kudzu (37.26 ± 0.82 g), pointed-leaf soybean (35.38 ± 1.31 g), and black locust (28.53 ± 0.81 g). When the larvae were fed on round-leaf soybean, they exhibited significantly higher survival rate (39.33 ± 0.90%), body weight (9.75 ± 0.07 g), total biomass (383.43 ± 7.35 g), pupation rate (87.78 ± 1.73%), and egg production (189.80 ± 1.06 eggs/female) compared to other hosts. These findings uncovered that round-leaf soybean significantly enhances larval performance, suggesting its potential for improving *C*. *bilineata* larval production and sustainability in cultivation systems.

## Introduction

Edible insects encompass those species that can be consumed directly by humans or used as feed for livestock, poultry, and fish [1]. Globally, there are over 3,650 identified edible insect species [2–4], with at least 1,400 of these being traditional culinary choices [5]. Commonly Commonly consumed orders include Isoptera, Orthoptera, Coleoptera, Hymenoptera, and Lepidoptera [6–8]. Termed as "21st-century foods", insects are highly regarded for their high protein and vitamin contents and low-fat profile [9–12], making them an increasingly popular choice worldwide. The Food and Agriculture Organization (FAO) recognizes insects as cost-effective and environmentally sustainable alternatives to traditional livestock such as pigs, cattle, and sheep, highlighting their potential to mitigate global food crises [13, 14]. Thus, the study and promotion of edible insects present significant opportunities for future research and development. *Clanis bilineata* (Lepidoptera, Sphingidae) [15] has recently gained recognition as a significant edible insect [16]. This species predominantly inhabits the Yellow and Huaihe River basins, the Yangtze River basin, and southern China. Its life cycle includes one generation per year in regions such as Shanxi, Hebei, Shandong, Jiangsu and Anhui; while in in areas like Hubei, Jiangxi and other provinces, it supports two generations annually [17–19]. In its natural habitat, *C*. *bilineata* primarily feeds on soybeans, black locust (*Robinia pseudoacacia*), kudzu (*Pueraria lobata*), and other plants. Historically, the larvae were considered aggressive pests of soybeans. They would hide on the underside of leaves by day and feed nocturnally, especially during the peak rainy months of July and August, leading to significant infestations [20]. However, the extensive and frequent application of insecticides has led to a sharp decline in their natural populations, reducing their impact on agriculture. Consequently, *C*. *bilineata* is now explored as a viable source of insect-based food [16].

In Jiangsu province and adjacent areas, the larvae of *C*. *bilineata*, locally “Doudan,” [21] are esteemed as a culinary delicacy. To prepare these larvae for consumption, the fifth instar larvae are initially soaked in water for 15 minutes. Subsequently, they are rolled from head to tail using a smooth cylindrical iron rod, measuring 3 cm in diameter and 25 cm in length. This method efficiently separates the skin from the internal contents. The extracted contents are then typically cooked with vegetables, yielding a dish that is both flavorful and nutritious [22].

Studies have demonstrated that *C*. *bilineata* larvae possess higher protein content, essential amino acids, and essential fatty acids than traditional protein sources like eggs, milk, and soybeans [23–27]. They also exhibit hypotensive and lipid-lowering properties, contributing to their growing popularity in traditional medicine for treating hypertension, coronary heart disease, and gastrointestinal disorders [17]. The culinary appeal of "Doudan" has spurred the development of numerous dishes and processed products, establishing a robust food culture with significant market scale and demand [28]. In 2021, the national production of artificially cultured larvae reached 3×10^4^ tons, with an output value of approximately 4.5 billion RMB (700 million USD). However, the market demand for the same year was estimated at 1×10^5^ tons [22], highlighting a substantial gap between production and consumer needs. Artificial production of *C*. *bilineata* is a relatively new venture. Currently, large-scale cultivation primarily utilizes pointed-leaf soybean as a food source, but yields of fifth-instar larvae remain low, typically ranging from 80 to 100 g/m^2^ [27]. Challenges such as lack of effective management in larval feeding, pupation, feathering, mating, egg laying, and hatching contribute to low production levels and inconsistent larval qualities [29, 30]. Recent studies focusing on improving hatchability through optimal preservation conditions and egg disinfection, as well as the impacts of different disinfectant formulas on egg growth and development, suggest potential strategies for enhancing artificial cultivation [28, 30]. There remains, however, an urgent need for systematic improvements in production technology to meet the rising demand and to fully harness the potential of *C*. *bilineata* as a sustainable food source.

While previous studies have primarily examined the nutritional quality and deep processing of *C*. *bilineata* [31–33], there has been limited research on its production efficiency and scalability. It is well-established that host plants significantly affect development, fecundity, and pupal weight in various insect species [34, 35]. Our preliminary observations in the field suggested that variations in host plants, such as differences in leaf shapes, might influence the biomass and quality of larvae. However, the specific impacts of various host species on the yield and quality of *C*. *bilineata* remain poorly understood.

To address this research gap, our study systematically evaluated the performance of *C*. *bilineata* larvae on different host plants, including round-leaf soybean, pointed-leaf soybean, black locust, and kudzu. We investigated the larvae’s feeding preferences and habits across these hosts and measured the effects of host plants on larval survival, body weight, biomass, pupation rate, fecundity, and egg production. The goal was to identify the optimal host plants to enhance the production efficiency and scalability of *C*. *bilineata*.

## Materials and methods

### Insect rearing

**Fig 1:**
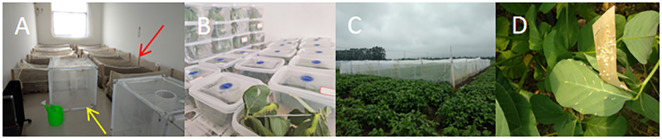
Rearing systems for *C*. *bilineata* in this study. (**A**) Environmental control room, featuring a distant rectangular wooden frame (red arrow) used for larval pupation and eclosion. The nearby cube cages (yellow arrow) were used for adult mating and oviposition. The heater and humidifier in the lower left corner control the temperature and humidity of the room. (**B**) Setup for indoor breeding of *C*. *bilineata*. (**C**) Field setup for large-scale production of *C*. *bilineata*. (**D**) The release of larvae in the field setup shows larvae that have just hatched on a host leaf.

Fifth instar larvae of *C*. *bilineata* were collected from soybean fields at the Dongsin Experimental Base of Lianyungang Academy of Agricultural Sciences, Lianyungang City, Jiangsu Province, China. The larvae were then transported to the laboratory for rearing. Wooden frames (2 x 1 x 0.3 m) were prepared, and disinfected soil with 75% moisture content was added to a depth of 20 cm wooden frame (Fig 1A). The larvae were maintained on fresh soybean leaves (positioned on the soil surface) under controlled conditions of 25°C and 75% RH. Leaves were replaced every two days. At the end of the fifth instar, as the larvae entered the diapause stage, they burrowed into the soil. The laboratory temperature was subsequently raised to 30°C, maintaining the same humidity levels. Following diapause, the larvae emerged to pupate, ascending to the soil surface, where their body color transitioned from light brown to dark brown. The temperature was then reduced back to 25°C, and approximately 15 days later, the pupae developed into adults. Adult females exhibited a light brown color, a thick abdomen, and an obtuse angle, while the males had a smaller abdomen with an acute angle. For mating, males and females were placed together in a 50 × 50 × 50 cm transparent, breathable plastic case. Eggs collected from these pairings were stored at 25°C to facilitate hatching.

### Odor selection test

**Fig 2:**
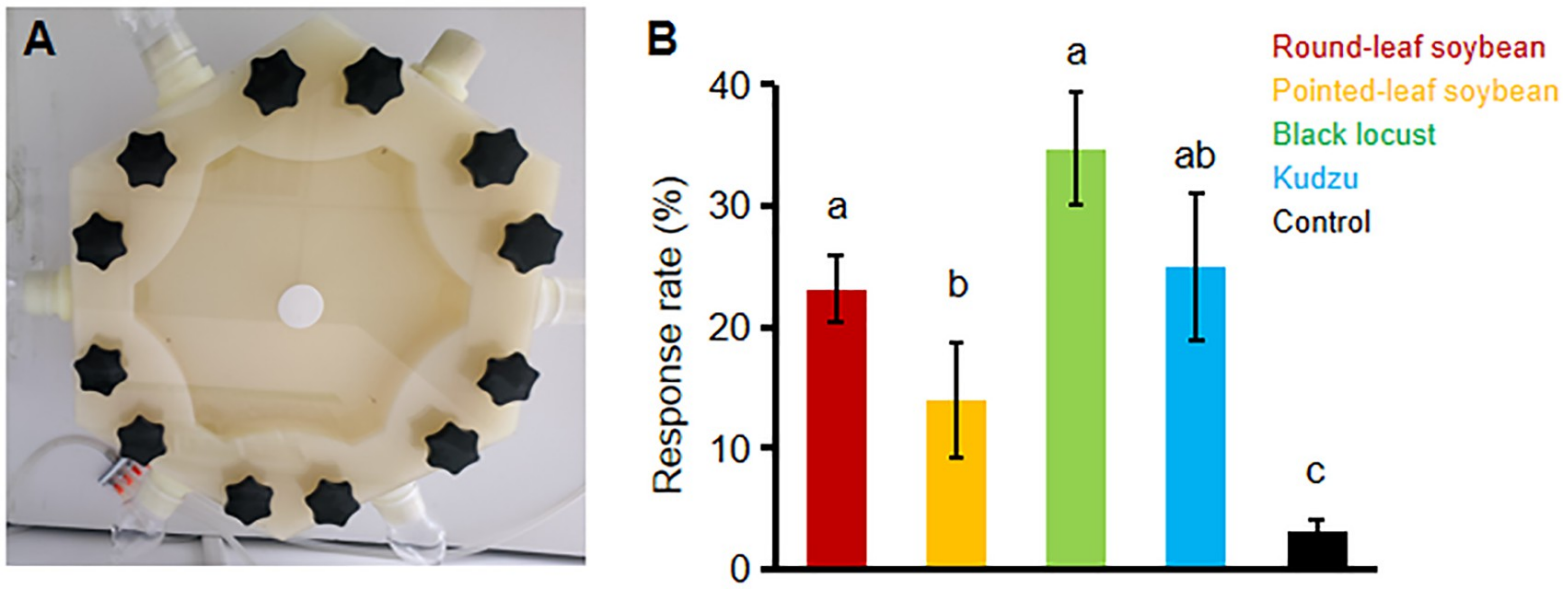
Odor selection of *C*. *bilineata* larvae to different host plants. (A) A picture of the insect’s six-arm olfactometer was used for the selection test. A total of 180 larvae were tested in a six-arm olfactometer. (B) The number of larvae lured in each of the four treatment and control groups was recorded and the selection response rate for each group was calculated. The lines on the bars represent standard errors (n = 60 per experiment, and the experiment was repeated three times). Significant differences between each group were tested using ANOVA, followed by Duncan’s new complex polar difference test. Different letters indicate significant differences at *P < 0*.*05*.

We evaluated the lure effects of four host plants (round-leaf soybean, pointed-leaf soybean, black locust, and kudzu) on *C*. *bilineata* larvae using a six-arm olfactometer (Beijing Xingyun Kenuo Glass Instrument Factory, Beijing, China; Fig 2A). This apparatus featured six arm inlets evenly distributed around a central cylindrical chamber, each with a diameter of 4 cm, and a singular air outlet at the base, measuring 0.5 cm in diameter. The central chamber, where the larvae were placed, had a 30 cm diameter and was 15 cm high, allowing free movement of the larvae. Prior to the selection test, air entering through the six inlets was first purified via activated carbon and then humidified after passing through a power pump and a water tower. The air subsequently flowed through pear-shaped bottles containing the odor sources and entered the larval activity chamber.

In the selection test, leaf cuts (~ 10 grams) of each host plant were placed in the pear-shaped bottles at inlets 1 to 4. The bottle at inlet 5 served as the control group and contained no leaves. The outlet at the bottom was sealed, and inlet 6 was used both as the entry point for the larvae and as an air outlet. The ambient conditions were maintained at 25°C with 75% humidity, and the airflow rate at each inlet was regulated to 400 ml/min. Once the system was stabilized, the third instar larvae of *C*. *bilineata* were individually introduced into the apparatus via inlet 6 using a soft brush. Larval activities were observed for 10 minutes. Larvae that failed to enter the activity chamber, or remained stationary upon entry were deemed to have made no selection. Conversely, larvae that moved toward any of the inlet 1 to 5 were recorded as having selected that specific odor source. The outcomes were analyzed using parameters defined by Li et al. [36]: total response rate (%) = (number of larvae that made a selection / number of larvae tested) × 100%; selection response rate = (number of larvae that selected an odor source/ number of larvae that made a selection) × 100%. Each larva was tested individually to avoid interference, with sixty larvae tested per experimental run, and the entire experiment was repeated three times.

### Determination of volatiles in host plant leaves

To collect volatiles, healthy and pest-free plants from each of the four hosts were selected. Targeted leaves were enclosed within glassware and sealed at the lower end with a glue plug and tin foil. This end was connected to a glass tube filled with activated carbon, while the upper end was attached to a 50 mg Porapak Q sorbent tube (Waters Associates Inc., Milford, USA). The sorbent tube was linked to the inlet of the atmospheric sampler (Qingdao Liheng Environmental Protection Equipment Co. Ltd., Qingdao, China), with the outlet connected back to the activated carbon tube, thus completing the gas circulation path. The atmospheric sampler operated at a flow rate of 1 L/min. After a continuous collection period of four hours, the adsorbed volatiles were eluted from the sorbent tube using 1 mL of HPLC-grade hexane. A 0.6 mL aliquot of this eluate was transferred into a 2 mL sample bottle and stored at a 4°C refrigerator. The volatile compounds were analyzed using a GC-MS (Shimadzu Corporation, Kyoto, Japan). The GC conditions employed a capillary column TG-5MS (30 m × 0.25 mm × 0.25 μm, 5% Phenyl Methylpolysiloxane) with an inlet temperature of 250°C. The temperature program began at 40°C, held for 3 minutes, then increased at 6°C/min to 160°C for 1 minute, and finally ramped at 20°C/min to 250°C for 5 minutes. The carrier gas used was high-purity nitrogen, delivered at a flow rate of 1.0 ml/min with no split injection and an injection volume of 1 μL. The MS conditions included a transmission line temperature of 280°C, quadrupole temperature of 150°C, ion source temperature of 280°C, ionization mode EI, and ionization energy of 70 eV. Full scan data were acquired over a mass range of 45–550 amu. Compound identification was performed using the NIST2.0 mass spectrometry with an automatic matching of mass spectral data and further verification by chromatographic retention times and reference standard spectra. The relative percentage of each component was calculated as (peak area of a single component/total area of volatiles) × 100%.

### Feeding assay

Newly hatched *C*. *bilineata* were reared in plastic cassettes, each measuring 25 x 15 x 15 cm and fitted with a lid (Fig 1B). To facilitate gas exchange while preventing larval escape, a 1 cm diameter air hole in the lid was covered with a breathable mesh. Inside each box, a 10 cm height wire was installed diagonally to suspend the leaves, which were attached using small clips to allow easy access for the larvae.

To ensure that the larvae had adequate food and that the leaves remained moist, the leaves in the boxes were sprayed every six hours and refreshed every two days. Each experiment started with ten newly hatched larvae per box, with leaves from each of the four host plants. Additional cages were set up under identical conditions to replace larvae lost to accidental deaths caused by operational errors or pathogen infections, with two such replacements occurring during the experiments.

Larvae feeding was monitored and recorded every two days. For the initial eight days, due to minimal larval consumption, feeding amounts were determined using grid statistics. Fresh and complete leaves from the four host species were weighed at five grams and outlined on 2 mm^2^ grid paper. The total number of grids covered by each leaf was counted to establish the leaf weight per unit grid area, facilitating the calculation of larval consumption. After two days of feeding, the leaves were reassessed against their initial outlines before feeding to determine the reduction in grid coverage, from which larval food intake was calculated using the following formula:

Larvalfoodintake=leafgridnumberreduced×leafweightperunitgrid.

Post the eight-day period, direct weighing of the leaves before and after feeding using a precision scale accurate to ten-thousandths was employed to ascertain consumption. The experiment lasted for 36 days until the larvae ceased feeding, and the total food intake for each host plant was calculated. This feeding experiment was conducted in three replicates.

### Effects of feeding on different hosts on field production of the larvae

In addition to the feeding assays conducted in the laboratory, we also examined the effect of the four host plants (round-leaf soybean, pointed-leaf soybean, black locust, and kudzu) on larval survival and growth under field production conditions. To accommodate the required number of larvae and pupae for the field, as well as the pupation and eclosion experiments, we established additional cages during the larvae feeding and rearing experiments. These cages were maintained under the same conditions as those established for each host plant, ensuring a consistent supply of insects for field experiments and the pupation and eclosion studies.

In the field, planting densities for the three hosts, including round-leaf soybean, pointed-leaf soybean, and kudzu, were calculated using the formula: row spacing × plant spacing = 30 × 15 cm. Each host was allocated to a treatment plotmeasuring 2 × 1 m (length × width), and each plot was enclosed by a net measuring 4 × 3 meters with 1 mm diameter holes that extended underground to prevent larval escape (Fig 1C). Three plots were utilized for each host to provide replicates.

For black locust, individual plants were pruned to form a hemisphere with a diameter of 1 meter, and each plant stood 1.5 meters high. Three such plants were employed, each serving as a separate replication. Newly hatched larvae were distributed evenly across the four treatment plots, with 100 larvae assigned to each plot or plant (Fig 1D). At the end of 30 days, which marked the conclusion of the fifth instar, the number of surviving larvae was recorded to determine the larval survival rate. Additionally, the total biomass of the surviving larvae was measured to calculate the average body weight.

### Effects of different hosts on different *C*. *bilineata* developmental stages

The fifth instar larvae that survived the field experiment were brought to the laboratory for further evaluation of their pupation rate, eclosion rate, and egg production. Throughout these laboratory experiments, the ambient conditions were controlled at 25°C with a relative humidity of 75%. For larval pupation and eclosion, a substrate consisting of a 1:1 mixture of soil and wood chips was prepared and placed into square wooden boxes approximately 20 cm thick with a relative humidity maintained at 70 ± 5%. Thirty fifth instar larvae from each host plant were segregated into four different wooden boxes to individually monitor pupation rates, with three replicates per host.

Additionally, a total of 90 pupae, derived from identical breeding and pupation conditions, were grouped into three replicates for an eclosion test. The eclosion outcomes for the four treatments were recorded daily. Upon the completion of eclosion, male and female adults were identified and separated. Subsequently, 20 male and 20 female adults from each treatment were randomly selected and placed into mating cages. After 8 hours, 10 pairs of mating or mated males and females were isolated into 10 plastic boxes to observe egg production, applying the same mating protocol across all four treatments.

### Data processing

In the host plant preference test, the differences between treated and control groups were analyzed using a chi-square test. The main parameters measured in the feeding assays included response rate, selection response rate, larval feeding, larval survival rate, body weight, total biomass, pupation rate, fecundity, and egg production. Data collected for all these parameters were statistically analyzed using SPSS v17.0, using one-way ANOVA followed by Duncan’s new complex polar difference method for multiple comparisons. Prior to these analyses, data pertaining to response rate, selection response rate, larval survival rate, pupation rate, and fecundity rate underwent an inverse arcsine square root transformation to stabilize variances and normalize distributions. Subsequently, normality was assessed using the Shapiro-Wilke tests before proceeding with statistical analyses.

## Results

### Host odor selection

In general, the larvae exhibited sensitivity to plant odors. In the host plant selection test using the six-armed olfactometer (Fig 2A), a significant majority of the larvae (i.e. 91.11%) demonstrated attraction to the odors presented, including the control. Notably, 96.95% of the larvae successfully identified and moved towards one of the four host plant leaves (Fig 2B). Among the four host plants, black locust elicited the highest selection response rate at 34.76%, followed by kudzu (25.00%), round-leaf soybean (23.17%), and pointed-leaf soybean (14.02%) (Fig 2B). Statistical analysis revealed no significant differences in the selection response rates among black locust, kudzu, and round-leaf soybean (*P > 0*.*05*). However, the selection response rate for the pointed-leaf soybean was significantly lower compared to that for black locust and round-leaf soybean (*P < 0*.*05)* (Fig 2B).

### Volatile components of the four host plant leaves

In total, we identified 20, 17, 20, and 19 volatile components from the leaves of round-leaf soybean, pointed-leaf soybean, black locust, and kudzu, respectively (Table 1). Specifically, 2,2,3,3,3-pentafluoropropyl acrylate, 2,2-dimethyl-3-heptanone, 2,2-dimethylbutane, 2,2,5-trimethylhexane, and tridecane were common across all four host plant species. The predominant volatile components in round-leaf soybean were 2,4-dimethyl-3-heptanone (14.75%), tridecane (14.71%), pentadecane (13.91%), and cis-butadienoic acid diallyl ester (10.66%). For pointed-leaf soybean, the main components were tridecane (39.12%), 2,2-dimethylbutane (15.58%), octanol (12.71%), and trans-1.5-heptadiene (4.90%). The main components of the volatiles from black locust leaf were 3,5-dimethylheptane (20.58%), tridecane (18.90%), vinyl acrylate (8.47%), and undecane (5.80%). The main components of the volatiles from kudzu leaf were undecane (45.74%), tridecane (17.28%), n-butylcyclohexane (6.64%), and methyl allyl ketone (3.84%).

**Table 1:** Composition of leaf volatiles of the four host plants.

Volatile Compounds		Relative amount (% mean ± standard error)			
		Round-leaf soybean	Pointed-leaf soybean	Black locust	Kudzu
Ketones	1,2-Benzisothiazol-3(2H)-one	0.56 ± 0.08			
	2(5H)-Furanone			0.23 ± 0.05	0.17 ± 0.02
	2,2-Dimethyl-3-heptanone	6.79 ± 1.11	5.78 ± 1.61	3.37 ± 0.27	2.86 ± 0.13
	2-Butanone	4.28 ± 0.52	2.95 ± 0.81		
	2-Coumaranone	1.61 ± 0.28	1.67 ± 0.52		
	2-Cyclohexen-1-one			1.30 ± 0.09	1.29 ± 0.20
	3-Heptanone, 2,4-dimethyl-	14.75 ± 2.34	4.39 ± 1.25		
	3-Nonone	5.01 ± 0.37			
	Decane, 2,2,4-trimethyl-			4.68 ± 0.71	3.84 ± 0.12
Esters	1H,1H-Pentafluoropropyl acrylate	0.70 ± 0.07	0.68 ± 0.22	0.69 ± 0.11	0.75 ± 0.05
	2(3H)-Furanone, dihydro-3-methylene-	0.38 ± 0.06	0.37 ± 0.10		
	2-Furanmethanol, tetrahydro-, acetate	0.12 ± 0.06	2.48 ± 0.68		0.21 ± 0.05
	Diallyl maleate	10.66 ± 1.59			
	Tri(propylene glycol) diacrylate	1.65 ± 0.23	1.46 ± 0.41	1.37 ± 0.63	
	2-Propenoic acid, ethenyl ester	1.44 ± 0.19	1.48 ± 0.20	8.47 ± 0.74	
Alkanes	2-Undecen-4-ol	9.75 ± 0.67	15.58 ± 0.80	1.08 ± 0.17	1.20 ± 0.07
	4-Penten-2-one			3.06 ± 0.18	2.86 ± 0.24
	Butane, 1,4-diiodo-	2.16 ± 0.35	1.91 ± 0.61		
	Cyclohexane, butyl-				6.64 ± 0.12
	Dodecane, 2,6,10-trimethyl-			2.19 ± 0.26	1.90 ± 0.03
	Heptane, 3,5-dimethyl-		1.27 ± 0.39	20.58 ± 8.04	
	Octane, 3,6-dimethyl-			5.00 ± 0.83	3.24 ± 0.57
	Pentadecane	13.91 ± 0.73		5.53 ± 0.49	
	Pentane, 2,2-dimethyl-		1.47 ± 0.41	2.16 ± 0.60	
	Pentane, 2-bromo-	3.29 ± 0.12			1.35 ± 1.16
	Pentane, 3,3-diethyl-	1.28 ± 0.07	1.53 ± 0.58	1.64 ± 0.22	0.68 ± 0.58
	Tridecane	14.71 ± 2.07	39.12 ± 1.91	18.90 ± 1.71	17.28 ± 0.49
	Undecane			5.80 ± 0.41	45.74 ± 0.83
	2,2,4,4-Tetramethyloctane			3.93 ± 1.47	2.64 ± 0.94
Other	Octanol	4.93 ± 0.74	12.71 ± 3.82		
	1,5-Heptadiene, (E)-	1.66 ± 0.10	4.90 ± 2.75		
	2-n-Butyl furan			1.96 ± 0.25	1.41 ± 0.17
	2-Pyrazoline, 5-ethyl-1,4-dimethyl-				0.64 ± 0.05
	Trans-1,2-Cyclopropanedicarboxylic acid			0.93 ± 0.13	0.97 ± 0.14

The composition of leaf volatiles varied significantly among the four host plants. The three most abundant volatile fractions in round-leaf soybean were alkanes (45.10%), ketones (33.01%), and esters (14.95%); while those of pointed-leaf soybean were alkanes (60.88%), ketones (14.79%), and alcohols (12.71%). The top three volatile fractions of black locust were alkanes (69.87%), esters (10.53%), and ketones (9.58%); while those of kudzu were alkanes (83.53%), ketones (8.16%), and acids (0.97%).

## Comparison of larval feeding on different host plants in the laboratory

**Fig 3:**
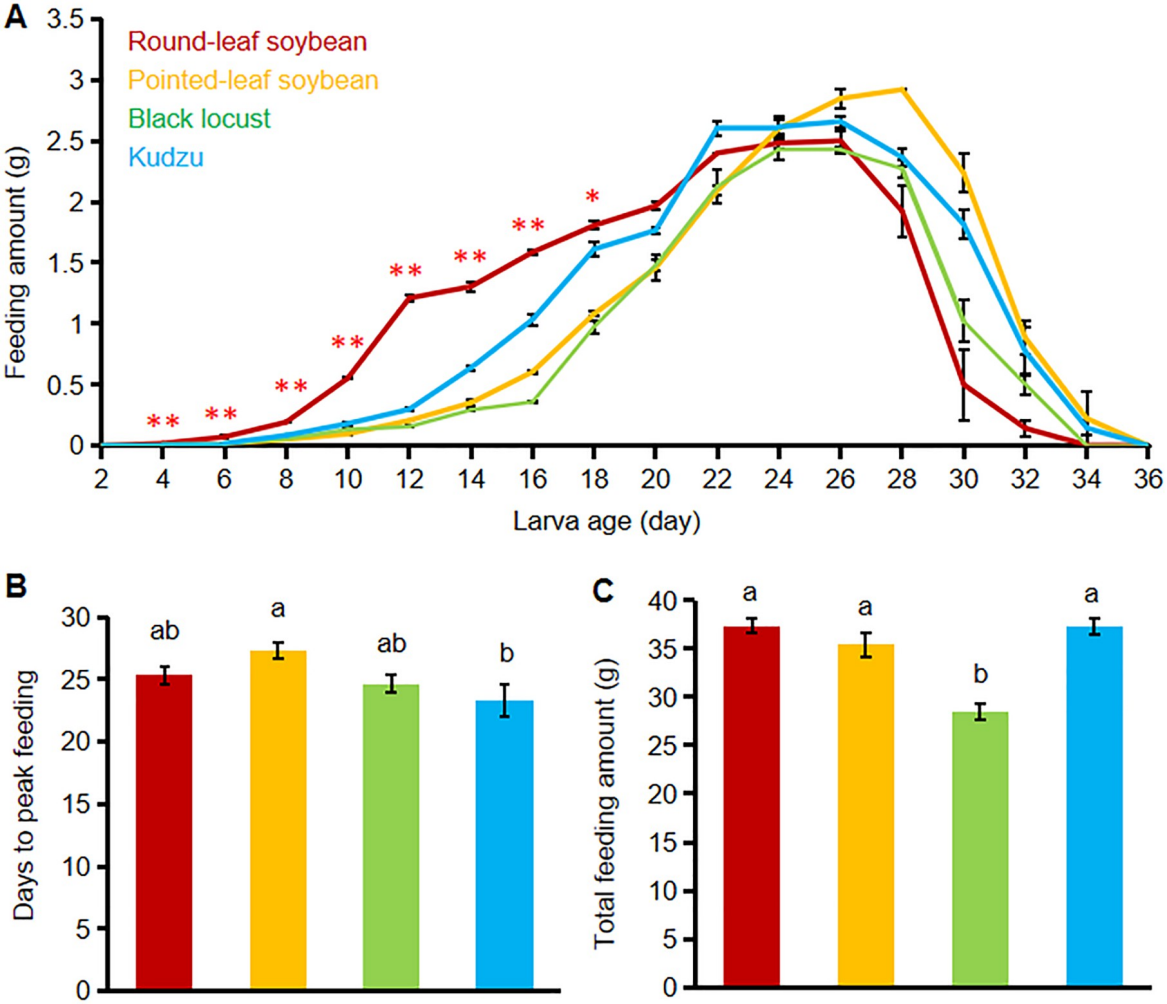
Comparison of larvae feeding on different host plants. (A) Larvae feeding on different host plants were measured every two days. (B) The number of days for the larvae to reach the peak feeding on different host plants. * (*P < 0*.*05*) and ** (*P < 0*.*01*) indicate significant differences between round-leaf soybean and the other three host plants. (C) The total feeding amount by larvae on different host plants across 36 days. Ten larvae were used for each host plant in each experiment (n = 10), and this experiment was repeated three times. Different letters on bars indicate significant differences at a *P < 0*.*05* tested using ANOVA, followed by Duncan’s new complex polar difference test.

The larval feeding experiments revealed significant variations in consumption depending on the host plant used (Fig 3). First, larvae fed on round-leaf soybean consumed significantly more leaf materials from day 4 to day 18, compared to larvae fed on the other three host plants (Fig 3A). Additionally, larvae on round-leaf soybean pupated two days earlier than those on the other host plants (34 vs. 36 days) (Fig 3A). Second, the timing to reach peak feeding also differed notably among the groups. Larvae feeding on round-leaf soybean reached their peak at approximately 25.33 days, similar to those on black locust (24.67 days) and kudzu (23.33 days), but significantly earlier than larvae on pointed-leaf soybean (27.33 days) (Fig 3B). The maximum daily feeding rates recorded were 2.50 g for round-leaf soybean, 2.92 g for pointed-leaf soybean, 2.43 g for black locust, and 2.66 g for kudzu. Third, the total consumption over the course of the experiment was 37.36 g for round-leaf soybean, 35.38 g for pointed-leaf soybean, 28.53 g for black locust, and 37.26 g for kudzu by an equal number of larvae. The total amounts consumed by larvae on round-leaf soybean, pointed-leaf soybean, and kudzu were not significantly different (*P > 0*.*05*); however, these were significantly higher than the total feeding amount on black locust (*P < 0*.*05*) (Fig 3C).

### Field culture of larvae on different host plants

**Fig 4:**
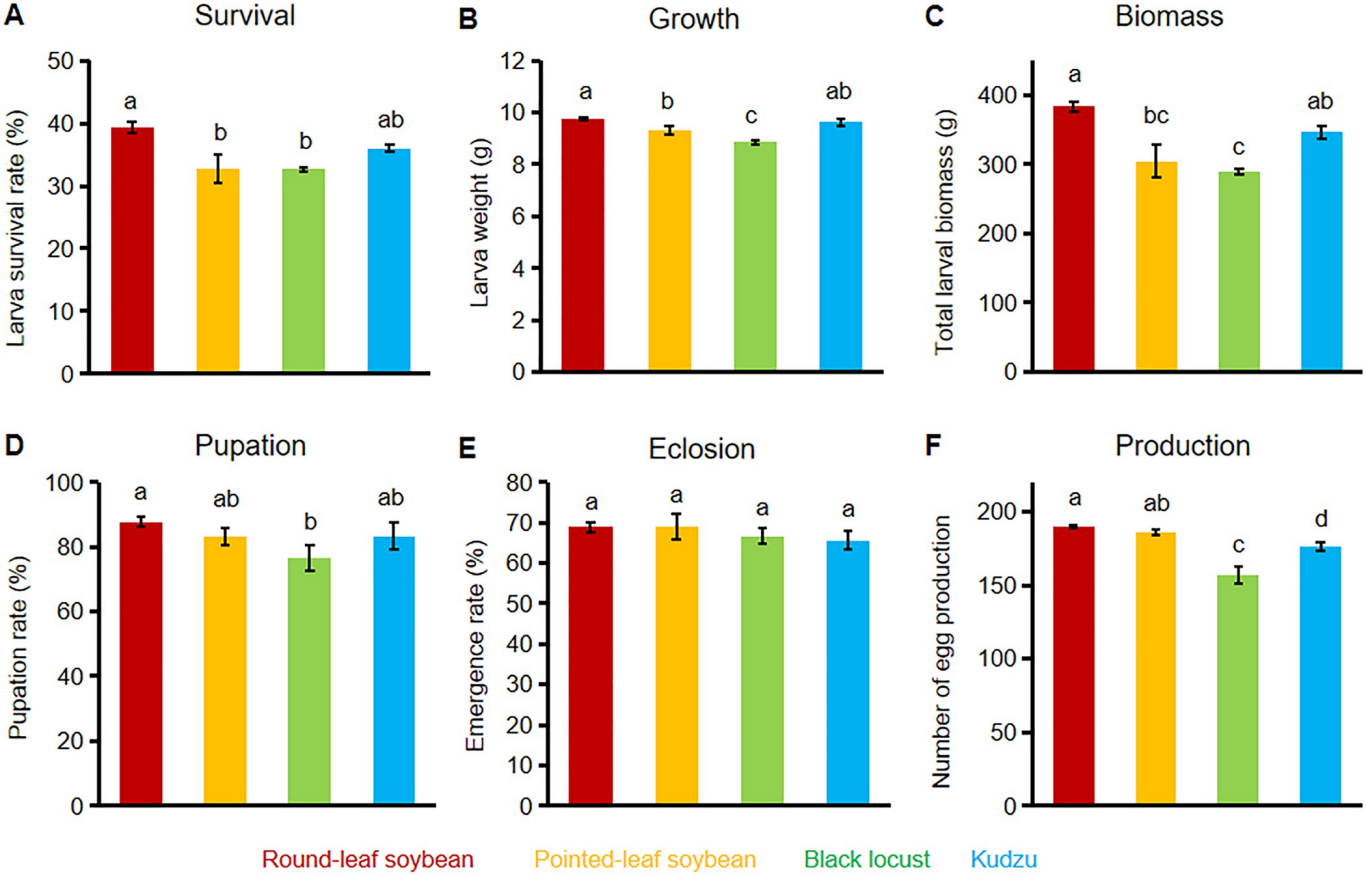
Effect of different host plants on the growth and development of field-raised larvae. (A, B, C) Bar graphs represent the survival rate (A), average body weight for growth (B), and the total biomass (C) of 5^th^ instar larvae. For panels A, B, and C, n = 100 for each group, and each group was repeated three times. (D, E, F) Bar graphs represent the effects of feeding on different host plants on the pupation rate (D) and eclosion rate (E) of larvae with n = 30 for each group and the egg production of female adults (F) with an n = 10 for each group. For panels D, E, and F, each group was repeated three times. Error bar = standard error. Different letters on bars indicate significant differences at *P < 0*.*05* tested using ANOVA, followed by Duncan’s new complex polar difference test.

In the field production of larvae, we monitored the larval survival, growth and total biomass. The highest survival rate of fifth-instar larvae was observed in round-leaf soybean treatments at 39.33%, followed by kudzu at 36.00%, and both pointed-leaf soybean and black locust at 32.67% each (Fig 4A). Larvae on round-leaf soybean and kudzu exhibited significantly higher survival rates compared to those on pointed-leaf soybean and black locust (*P < 0*.*05*). Regarding the average body weight (Fig 4B) and total biomass (Fig 4C) of the fifth instar larvae, round-leaf soybean led with 9.75 g and 383.43 g, respectively, followed by kudzu (9.63 g and 346.61 g), pointed-leaf soybean (9.32 g and 304.84 g), and black locust (8.87 g and 289.78 g). The larvae reared on round-leaf soybean exhibited significantly higher larval weight and total biomass compared to those reared on pointed-leaf soybean and black locust (*P < 0*.*05*). However, these values were not significantly different from those reared on kudzu (*P > 0*.*05*).

### Effects of different host plants on the development of *C*. *bilineata*

The surviving fifth instar larvae from the field experiments were transferred to the laboratory for further monitoring of their pupation rate, eclosion rate, and egg production (Fig 4D–4F). The highest pupation rate was observed in larvae fed on round-leaf soybean at 87.78%, followed by 83.33% for both pointed-leaf soybean and kudzu, and 76.67% for black locust. The pupation rate of *C*. *bilineata* on round-leaf soybean was significantly higher than that on black locust (*P < 0*.*05*), but there were no significant differences compared to those on pointed-leaf soybean and kudzu (*P > 0*.*05*) (Fig 4D). The eclosion rates for *C*. *bilineata* were similar across all host plants: 68.89% for both round-leaf and pointed-leaf soybean, 66.67% for black locust, and 65.56% for kudzu, with no significant differences noted (*P > 0*.*05*) (Fig 4E). Egg production varied significantly among the different host plants. On average, females raised on round-leaf soybean produced the highest average number of eggs at 189.80 eggs per female, followed by pointed-leaf soybean at 185.93 eggs per female, kudzu at 176.06 eggs per female, and black locust at 156.93 eggs per female (Fig 4F). There was no significant difference in egg production between females raised on round-leaf soybean and pointed-leaf soybean (*P > 0*.*05*); however, their productivity was significantly higher (*P < 0*.*05*) than those on black locust and kudzu (Fig 4F).

## Discussion

In this study, we systematically explored the effect of four host plants (round-leaf soybean, pointed-leaf soybean, black locust, and kudzu) on the growth and development of *C*. *bilineata*, an economically significant and edible insect. We first determined the larval host plant preference and their subsequent feeding behavior on the four host plants in the laboratory. Additionally, we examined how these four host plants influenced the insects’ survival rate, body weight, total biomass, pupation rate, eclosion rate, and egg production under field settings (S1 Table). Overall, our findings suggested that round-leaf soybean was the most suitable host for cultivating *C*. *bilineata* larvae. Given the current cultivation area for *C*. *bilineata* larvae in China remains constant, adopting round-leaf soybean could substantially enhance the total market supply of larvae.

Host plants significantly influence the behavior of insects, such as positioning, feeding, and egg-laying, primarily through the emission of specific volatiles [37–40]. In our study, all four selected host plants—round-leaf soybean, pointed-leaf soybean, black locust, and kudzu—effectively attracted the larvae. Notably, the larvae in this test exhibited higher selection response rates to the leaves of black locust, round-leaf soybean and kudzu than to pointed-leaf soybean (Fig 2). Analysis of the volatiles of leaves revealed that alkanes and ketones were major components common to all four host plants (Table 1). However, differences were observed: black locust,round-leaf soybean and kudzu predominantly contained esters and acids, whereas pointed-leaf soybean contained alcohols (Table 1). These findings suggest potential explanations for the observed selection responses: (1) Esters and acids in the leaves of black locust, round-leaf soybean and kudzu may attract larvae. (2) Conversely, alcohol compounds in the leaf of pointed-leaf soybean may repel larvae. (3) A combination effect from both scenarios (1) and (2) may influence larval preferences. Previous research indicates that low concentrations of plant volatiles can lure target insects, while higher concentrations might not produce a luring effect [41]. Thus, the differences in the concentrations of trace volatiles in leaves could also explain the differential selection responses among the larvae to the four host plants. Further studies are required to examine the lure effects of all identified volatiles in these host plants, to determine the effective attractant compounds and to elucidate the mechanisms underlying larval preference for host plant odors.

While a preference for host plant odors may indicate potential feeding sites, it does not necessarily translate into actual feeding behavior. For example, a study on the chafer beetle (*Holotrichia oblita*) demonstrated a higher olfactory attraction to castor than elm, yet the beetles exhibited significantly lower feeding on elms [42]. This discrepancy between odor preference and feeding behavior was also observed in our study; although *C*. *bilineata* larvae showed a higher selection response rate for black locust compared to pointed-leaf soybean (Fig 2B), their actual feeding on black locust was significantly less (Fig 3C). Such variations in feeding can directly impact larval body weight, total biomass, and ultimately survival rates. Enhancing leaf consumption is a viable method for increasing larval biomass per unit area in production settings. The specific nutrients in host leaves that most significantly affect larval feeding preferences remain unexplored in this paper. This is a crucial aspect of our experimental approach that warrants further investigation. Additionally, the limited number of treatments and replicates in this experiment provides guidance primarily for small-scale breeding, and its applicability to large-scale farming remains to be established. Future research will involve conducting a series of behavioral tests to identify the specific nutrients (e.g. sugars, esters, and proteins) that significantly enhance larval food intake. The aim will be to improve larval culture efficiency by applying these nutrients directly to the host leaves. Moreover, to build on the methodology of this study, we plan to expand the sample size of each experiment and re-analyze the data to validate these findings for large-scale breeding of *C*. *bilineata*.

In the cultivation of *C*. *bilineata* larvae, soybeans and kudzu have commonly served as host plants, with established planting methods and pest and disease management practices already in place [43]. However, these established methods have primarily reported on larval survival rate and yield per unit area, with limited information on population rates and egg production. Our study found that larval fed on round-leaf soybean exhibited higher survival, pupation rate, and egg production compared to those fed on the other three host plants (Figs 3 and 4). While the existing planting methods yield a larval survival rate of 28.00% [43], the use of round-leaf soybean as a host improved the survival rate to 39.33%. Furthermore, larvae raised on round-leaf soybean showed enhanced growth and development, resulting in a higher yield of larval biomass of 191.71 g/m^2^. Compared to the current yield of 134.93 g/m^2^ from pointed-leaf soybean [43], round-leaf soybean proves to be a more viable host for the commercial production of *C*. *bilineata*. Additionally, it has been reported that round-leaf soybean can still produce a significant yield of soybeans after being used as a host plant in *C*. *bilineata* production, thereby increasing the economic returns per unit area of land [44].

Additionally, it has been documented that insect feeding can trigger defensive responses from host plants [45–48]. In the field culture, feeding by *C*. *bilineata* larvae often results in the emergence of host plant pests such as bridge worms and bean pyralid (*Lamprosema indicata* Fabricius) [43]. An intriguing observation from our field studies of *C*. *bilineata* larvae showed that when soybean leaves were partially consumed by these pests, the larvae frequently ceased climbing and refused to feed on the infected and adjacent leaves. This behavior is currently not well-documented in the literature. Drawing from two previous studies on plant defense mechanisms [49, 50], we hypothesize that pest feeding might activate the plant’s defense systems, leading to physiological and biochemical changes (e.g. metabolic processes) that subsequently produce compounds repellent to *C*. *bilineata* larvae. Further research is necessary to elucidate these defensive cascades and their impact on larval behavior. Additionally, competition with other pest species could also contribute to the observed low and non-sustainable populations of *C*. *bilineata*. Further studies should aim to explore the interactions between *C*. *bilineata* and other pest species on soybeans to better understand the underlying mechanisms influencing these dynamics.

Beyond the challenges of population variability and environmental constraints in the field, the artificial breeding and larval production of *C*. *bilineata* remain relatively nascent practices, confronting numerous technological challenges. Nevertheless, significant advancements have been made. For instance, eggs are now treated with specific disinfectants to eliminate harmful microorganisms, thereby maintaining a high hatching rate [28]. We have also explored the effects of varying egg densities on larval survival rate, weight of mature larvae and total biological yield under a consistent planting density of soybean, establishing an economic threshold for egg density [44]. However, several issues continue to impede the efficient production of *C*. *bilineata*. Currently, the larvae rely solely on live soybeans and kudzu as food sources, with no artificial diet yet developed. Creating an artificial diet is crucial for overcoming the limitations posed by the need for cultivating host plants in the field and would facilitate efficient, intensive, and year-round breeding [51]. Additionally, the natural cycle of *C*. *bilineata* in Jiangsu Province, China, typically involves a single generation per year, with larvae entering dormancy from November to April [28, 43]. As market demands exceed the output from a single annual generation, research efforts have aimed to circumvent larval dormancy and enable multi-generation production [52]. Yet, maintaining high larvae activity post-dormancy interruption remains a substantial challenge. Furthermore, breeding companies currently rely on a limited number of artificially cultured *C*. *bilineata* populations for succession breeding. After several generations, these artificially bred populations often exhibit reduced resilience, high mortality, and diminished fertility due to the lack of external gene exchange [16]. Addressing the issues related to the artificial breeding populations of *C*. *bilineata* and overcoming technical bottlenecks related to dormancy release are critical for meeting market demands and enhancing the viability of the *C*. *bilineata* breeding industry.

## Conclusion

In this study, we systematically evaluated the impact of different host plants on various parameters of *C*. *bilineata* larval, including survival rate, average body weight, and biological yield. Our findings revealed that larvae exhibited optimal performance on round-leaf soybean, achieving the highest biomass yield and the greatest number of eggs for successive breeding, highlighting round-leaf soybean as the most suitable host plant for *C*. *bilineata* production. The insights gained from this research not only provide a critical theoretical foundation for understanding the growth and reproduction of *C*. *bilineata* and their interactions with host plants, but also pave the way for advancing the technology of artificial *C*. *bilineata* production. This endeavor aims to broaden the utilization of this emerging and locally popular edible insect on a global scale, thereby enhancing human food security.

## Supporting information

S1 Table The data in Figs 2–4 are shown in Tables A-D of S1 Table. (DOCX)
