# Supplementary material for: Selection of host plants for production of Clanis bilineata (Lepidoptera: Sphingidae)
Source: PLoS One. 2024 Jun 24;19(6):e0303017. doi: 10.1371/journal.pone.0303017 (PMC11195959; doi:10.1371/journal.pone.0303017)
Supplement: S1 Table — (DOCX) [file pone.0303017.s001.docx]

**Table A** Odor selection of *C. bilineata* larvae to different host plants

| Species of hosts | Response rate/% | Selection response rate/% |
| --- | --- | --- |
| Round-leaf soybean | 91.11±0.56 | 23.17±2.79 ab |
| Pointed-leaf soybean |  | 14.02±4.74 b |
| Black locust |  | 34.76±4.65 a |
| Kudzu |  | 25.00±6.12 ab |

**Table B** Comparison of larvae feeding on different host plants

| Day age of larva (day) | Round-leaf soybean | Pointed-leaf soybean | Black locust | Kudzu |
| --- | --- | --- | --- | --- |
|  | Feeding amount (g) | | | |
| 2 | 0.002075227 | 0.001897788 | 0.001146186 | 0.002070097 |
| 4 | 0.019358903 | 0.00783603 | 0.006533258 | 0.006924119 |
| 6 | 0.072721882 | 0.015243527 | 0.019493116 | 0.017944809 |
| 8 | 0.194189363 | 0.053872705 | 0.054462922 | 0.085642204 |
| 10 | 0.55360138 | 0.097056139 | 0.13353063 | 0.181668887 |
| 12 | 1.211725026 | 0.207517778 | 0.156225106 | 0.299521669 |
| 14 | 1.305248588 | 0.352621344 | 0.293882005 | 0.639017632 |
| 16 | 1.587237345 | 0.606271998 | 0.359558444 | 1.035619728 |
| 18 | 1.806485074 | 1.081739436 | 0.975404002 | 1.612391675 |
| 20 | 1.967 | 1.458666667 | 1.481333333 | 1.767333333 |
| 22 | 2.400333333 | 2.092666667 | 2.127333333 | 2.606333333 |
| 24 | 2.482666667 | 2.602 | 2.430666667 | 2.616666667 |
| 26 | 2.500666667 | 2.848333333 | 2.427 | 2.659333333 |
| 28 | 1.924 | 2.921333333 | 2.272333333 | 2.366 |
| 30 | 0.502 | 2.236 | 1.020333333 | 1.815333333 |
| 32 | 0.143333333 | 0.885 | 0.503333333 | 0.771666667 |
| 34 | 0.005 | 0.223333333 | 0 | 0.145 |
| 36 | 0 | 0 | 0 | 0 |

**Table C** Effect of different hosts on the growth and development of field-raised larvae

| Hosts | Survival rate of larvae /% | Weight of larva /g | Total larval biomass /g |
| --- | --- | --- | --- |
| Round-leaf soybean | 39.33±0.90 a | 9.75±0.07 a | 383.43±7.35 a |
| Pointed-leaf soybean | 32.67±2.32 b | 9.32±0.17 b | 304.84±23.72 bc |
| Black locust | 32.67±0.36 b | 8.87±0.08 c | 289.78±4.83 c |
| Kudzu | 36.00±0.60 ab | 9.63±0.14 ab | 346.61±8.58 ab |

**Table D** Effect of consumption of different hosts on the development of different stage of *C. bilineata*

| Hosts | Pupation rate /% | Emergence rate % | Egg production |
| --- | --- | --- | --- |
| Round-leaf soybean | 87.78±1.73 a | 68.89±1.19 a | 189.80±1.06 a |
| Pointed-leaf soybean | 83.33±2.59 ab | 68.89±3.16 a | 185.93±1.92 ab |
| Black locust | 76.67±3.87 b | 66.67±2.04 a | 156.93±5.99 c |
| Kudzu | 83.33±4.34 ab | 65.56±2.36 a | 176.06±3.09 b |
